# A Latent Dirichlet Allocation approach to understanding students’ perceptions of Automated Writing Evaluation

**DOI:** 10.1016/j.caeo.2024.100194

**Published:** 2024-06

**Authors:** Joshua Wilson, Saimou Zhang, Corey Palermo, Tania Cruz Cordero, Fan Zhang, Matthew C. Myers, Andrew Potter, Halley Eacker, Jessica Coles

**Affiliations:** a School of Education, University of Delaware, 213E Willard Hall Education Building, Newark, DE 19716, United States; b Measurement Incorporated, United States; c Arizona State University, United States

**Keywords:** Automated Writing Evaluation, Automated feedback, Feedback, Latent Dirichlet Allocation, LDA, Perceptions

## Abstract

Automated writing evaluation (AWE) has shown promise in enhancing students’ writing outcomes. However, further research is needed to understand how AWE is perceived by middle school students in the United States, as they have received less attention in this field. This study investigated U.S. middle school students’ perceptions of the *MI Write* AWE system. Students reported their perceptions of MI Write's usefulness using Likert-scale items and an open-ended survey question. We used Latent Dirichlet Allocation (LDA) to identify latent topics in students’ comments, followed by qualitative analysis to interpret the themes related to those topics. We then examined whether these themes differed among students who agreed or disagreed that MI Write was a useful learning tool. The LDA analysis revealed four latent topics: (1) students desire more in-depth feedback, (2) students desire an enhanced user experience, (3) students value MI Write as a learning tool but desire greater personalization, and (4) students desire increased fairness in automated scoring. The distribution of these topics varied based on students’ ratings of MI Write's usefulness, with Topic 1 more prevalent among students who generally did not find MI Write useful and Topic 3 more prominent among those who found MI Write useful. Our findings contribute to the enhancement and implementation of AWE systems, guide future AWE technology development, and highlight the efficacy of LDA in uncovering latent topics and patterns within textual data to explore students’ perspectives of AWE.

## 1 Introduction

Writing involves coordinating cognitive, metacognitive, and affective processes, extending beyond the mere act of transcription [1]. Writing is therefore complex, and many students in the United States struggle to demonstrate writing proficiency [2]. Accordingly, researchers are focusing on developing and validating innovative instructional methods that enhance students’ abilities to compose well-organized and elaborated texts that demonstrate command of written language and its conventions (e.g., [3]). Educational technology applications have proven to be effective in this regard [4].

Automated writing evaluation (AWE), which provides students with immediate evaluative scores and feedback through software applications [[5], [6], [7]], has emerged as one such promising method. The adoption and utilization of AWE has increased in recent years [8,9], presenting a unique opportunity to enhance writing instruction. While studies evaluating the effectiveness of AWE have generally shown positive outcomes [[6], [7], [10]], it is equally important to understand how AWE is viewed by its primary beneficiaries, the students who use it [11].

### 1.1 Student perceptions of AWE

Students of various educational levels generally view AWE systems positively as effective tools that promote their writing practice [[12], [13], [14], [15]]. For example, in one study involving a districtwide implementation of the *MI Write* AWE system, students in Grades 3–5 responded to a survey of Likert scale items and reported positive perceptions regarding the ease of using MI Write (usability), the benefits it provided (usefulness), and their individual inclination to use MI Write again or recommend it to their peers (desirability) [13]. Notably, students most strongly endorsed MI Write's usability, as well as its usefulness for identifying areas of improvement and aiding them in revising.

One factor that appears to influence students’ holistic view of AWE is how they perceive the accuracy and usefulness of AWE's automated feedback. Research examining EFL undergraduates utilizing automated written corrective feedback revealed that when compared to teacher feedback, automated feedback identified a lower number of errors in students’ writing (i.e., exhibited reduced recall). Additionally, the precision of AWE in accurately diagnosing the specific errors was found to be relatively lower [16,17]. For example, in a study conducted by Li et al. [18] with 135 mid and lower-level university ESL students in the United States, most students considered AWE feedback from *Criterion* to be beneficial, particularly the grammar feedback. However, nearly half had concerns about the clarity of Criterion's feedback for improving specific grammatical errors such as run-on sentences. Consequently, in the presence of such inaccuracies, even when students maintain positive perceptions of AWE, they tend to express a preference for teacher feedback [17].

To further illustrate, Bai and Hu [16] studied the *Pigai* AWE system in the context of a university-level EFL English course with the 30 sophomores. The authors specifically examined Pigai's feedback related to synonyms and collocations. The authors found that the accuracy of the synonym feedback was high (94.03 %), and the majority (60 %) of university EFL participants perceived it as useful. However, the quality of the revisions students made based on these suggestions was relatively low. This discrepancy can be attributed to the fact that the synonym feedback generated by Pigai lacked sufficient comparison or explanation, resulting in learners’ indiscriminate use of synonyms and subsequent lexical errors. Conversely, results revealed strikingly low accuracy of collocations feedback (21.83 %), resulting in only 40 % of students trusting Pigai's feedback in this area. These findings illustrate the challenges associated with AWE systems in providing accurate and effective feedback to improve students’ lexical proficiency and the associated influence on students’ perceptions of AWE.

The specificity of automated feedback is another factor that influences students’ perceptions of AWE. Ranalli [19] discovered that undergraduate ESL students (*n* = 82) perceived specific feedback as easier to comprehend and less mentally demanding than generic feedback. Furthermore, students exhibited a five-fold increase in accurate corrections when provided with specific rather than general feedback. These findings align with those of Zhu et al. [20], which also demonstrated the advantage of specific automated feedback over generic feedback in facilitating US secondary students’ acquisition of scientific argumentation writing skills. Regardless, as illustrated in Li et al.’s [21] examination of AWE with 70 undergraduate ESL students, AWE feedback tends to be too generic and thus difficult to implement. For example, Moore and MacArthur [22] explored the use of the *MY Access!* AWE system by six middle-school students for two writing tasks. Three distinct groups of students emerged: those who utilized and acted upon the AWE feedback, those who ignored the feedback, and those who reviewed the feedback but struggled to apply it effectively. Even the students who used the feedback faced difficulties in understanding and incorporating it, producing surface-level edits rather than significant revision improvements.

When it comes to providing specific feedback on higher-level writing skills such as content, organization, and genre, AWE feedback tends to be limited (see [23,24]), although efforts are underway to advance AWE's capabilities in this regard (see [25,26]) and results are quite encouraging (see [27]). Accordingly, findings from prior research indicate that students tend to prefer higher-level feedback from humans versus AWE [15] and implement human feedback to a greater extent than AWE feedback [24].

Finally, perhaps given the inconsistent record of automated feedback's accuracy, specificity, and utility, students’ perceptions regarding the benefits of AWE for enhancing their writing motivation have been mixed. On one hand, Grimes and Warschauer [14] reported that nearly two thirds of middle school students surveyed expressed greater motivation to write and revise with the support of the MY Access! AWE system. Also, Ware [15] found that Criterion elicited a sense of enjoyment among users, fostering increased interest and engagement in the writing process. On the other hand, Wilson and Czik [12] observed that although students using MI Write reported greater persistence solving problems in their writing, their overall disposition towards writing remained unchanged. Similarly, in Wilson et al.'s [13] study involving upper-elementary students, students surveyed tended not to agree that MI Write increased their writing motivation.

In sum, students generally hold positive perceptions of AWE systems as effective tools for promoting writing practice, particularly in terms of usability and usefulness for identifying areas of improvement. However, students’ desire to continue using AWE systems in the long term appears to be relatively lower, and students’ perceptions of AWE's benefits for enhancing writing motivation are mixed. The accuracy, specificity, and utility of AWE's automated feedback play a significant role in shaping students’ perceptions, with studies highlighting challenges in providing accurate and effective feedback. Additionally, the limited provision of specific feedback on higher-level writing skills and the preference for human feedback indicate ongoing areas for improvement in AWE systems.

However, there remain several notable gaps in the literature that has examined students’ perceptions of AWE. First, relative to research examining AWE's impact on student outcomes, there is much less research on student perceptions of AWE. Further, of that research, little has focused on U.S. middle school students. Much of the extant research involves undergraduate EFL or ESL learners who experience different instructional and motivational contexts for learning to write. Similarly, while seminal early work involved U.S. middle school populations (e.g., [14]) recent advancements in AWE technology and increases in technology-mediated learning warrants further exploration because students may now hold more critical and nuanced perceptions of educational technology applications than a decade (or more) ago. Finally, more research is needed to understand how students perceive AWE and to identify common, salient, and generalizable themes in their user experiences. Along those lines, there is a need for methodological innovation in this area of research. Prior research probing student perceptions has either employed surveys, which offer students limited opportunities to share nuanced perceptions, and/or interviews and focus groups conducted with small samples and analyzed qualitatively, making it difficult to generalize findings to broader populations. Advances in computational modeling have resulted in new analytic techniques that reduce the time and resources required of traditional qualitative analysis and thereby allow for examining the nuanced perceptions of much larger samples of students. Importantly, these new methods provide a replicable framework that can be applied across various studies and contexts.

### 1.2 Study purpose

The present study addresses these aforementioned gaps in the literature on students’ perceptions of AWE by investigating the perspectives of a diverse sample of U.S. middle school students regarding the AWE system MI Write. We employ a novel analytic approach—latent Dirichlet allocation (LDA)—to uncover hidden topics and themes in students’ written comments regarding their perspectives on and preferences for AWE features and functionality. LDA, a computational modeling technique used in natural language processing, allows for identifying latent themes within a corpus of text data [28,29]. For instance, LDA has been used productively to identify latent research interests and research trends in published articles on topics such as the flipped classroom [30], computational thinking [31], and educational data mining [32]. In a similar way, by applying LDA to students’ written responses regarding their experiences with AWE, we gain insights into their perceptions, preferences, and challenges related to the software, and do so in a way that supports the generalizability of study findings. The present study, thus, not only addresses the limited understanding of students’ perceptions of AWE in the existing literature but also provides a demonstration of the affordances of LDA for research of this kind.

We applied LDA and subsequent mixed methods analyses to answer the following research questions:

  (1) What main themes about MI Write are revealed from students’ comments?
  (2) Do these themes differ based on how useful students perceive MI Write to be?

## 2 Methods

### 2.1 Study context

The present study examines comments provided by students in Grades 7 and 8 who were given access to an AWE system called MI Write in the context of a randomized controlled trial (RCT). Conducted during the 2021–2022 school year, the RCT examined the efficacy of MI Write on students’ writing outcomes (see [33]).

MI Write (www.miwrite.net) is an AWE tool developed and marketed by Measurement Incorporated. The tool is designed to assist writing instruction and learning by offering immediate, automated feedback and scores in response to student writing [34]. At the time of the RCT, MI Write leveraged the automated essay scoring system *Project Essay Grade* (PEG; [[35], [36], [37]]). PEG provided students with an automated writing-quality score ranging from 6.0 to 30.0, as well as scores for six distinct writing traits, including idea development, organization, style, sentence fluency, word choice, and conventions, based on the Six Trait Model [38]. Using the Six Trait scoring model, MI Write provided trait-specific feedback and suggestions for improvement, along with metacognitive prompts to help students evaluate their writing, such as checking for a clear conclusion. In addition, MI Write offered plagiarism detection and in-line feedback regarding spelling and grammar errors. MI Write also included multimedia lessons that aligned with students’ writing performance and peer review functionality. Teachers could customize prompts, communicate with students through messages, and provide additional feedback by using in-line and summary comments in response to student writing.

The RCT involved three school districts in the Mid-Atlantic and Southern regions of the United States. Teachers were randomly assigned in blocks to either the MI Write treatment condition (*n* = 19) or a business-as-usual comparison condition (*n* = 18)—assignment blocks were determined by their district, school, and grade level. School districts provided the research team with demographic data on all students. The research team collected survey and writing performance data electronically via Qualtrics at baseline and follow-up in September and late May/early June, respectively. The student perceptions data used in this study were only collected in the spring (see Measures section). The study was conducted with institutional board review approval.

### 2.2 Sample

A total of 1299 students were randomly assigned to the intervention group and had access to MI Write. Of these students, 981 students provided a response to an optional open-ended item probing their perceptions of MI Write. The analytic sample consisted of the comments from 889 students that were retained after data preprocessing (see Data Analysis section). None of these students had prior experience using MI Write.

A comparison of the demographics of the analytic sample with the original (i.e., full) sample of students in the intervention group is presented in Table 1. There were no statistically significant differences in the demographics of these samples for grade (*χ*2 = 0.90, *df =* 1*, p* = .342), sex (*χ*2 = 0.16, *df* = 1, *p* = .692), race (*χ*2 = 7.69, *df* = 4, *p* = .104), special education status (*χ*2 = 2.51, *df* = 1, *p* = .113), or limited English proficiency status (*χ*2 = 3.13, *df* = 1, *p* = .077), but there were significant differences for free/reduced lunch status (*χ*2 = 4.28, *df* = 1, *p* = .039) and district (*χ*2 = 6.62, *df* = 2, *p* = .037). Overall, this indicates that the analytic sample generally represented the full sample of students provided with access to MI Write with the exception of a slightly smaller proportion of students who received free/reduced lunch (4.0 % difference), a slightly higher proportion of students from District 1 (4 % difference), and a lower proportion from District 2 (3 % difference). The analytic sample was demographically diverse as indicated in Table 1.

**Table 1:** Demographics of the original (full) sample and analytic sample of students.

	Full Sample (*n* = 1299)	Analytic Sample (*n* = 889)
District		
1	491 (38 %)	373 (42 %)
2	565 (43 %)	338 (38 %)
3	243 (19 %)	178 (20 %
Grade		
7	288	182
8	1011	707
Sex		
Female	659	443
Male	641	446
Race		
White	329	243
Hispanic	558	386
Black	236	127
Asian	122	100
American Indian or Pacific Islander	11	8
Multiracial	43	25
Free/reduced lunch recipient	824	525
Special education recipient	115	62
Limited English proficiency	50	22

### 2.3 Measures

The present study examines student responses to the optional open-ended item administered to intervention students in the spring. The item asked, *“What would you like the MI Write creators to know about how the program can better support students like you?”* and was intended to probe students’ perceptions about MI Write's strengths and areas of improvement.

In addition, the present study uses data from the usefulness subscale of the student version of the *AWE Perceptions Scale* ([39]). The usefulness subscale comprised six items, each rated on a Likert scale from 0 (strongly disagree) to 3 (strongly agree): (1) *MI Write helped me plan my writing*; (2) *MI Write helped me revise my writing*; (3) *MI Write helped me learn more about writing*; (4) *MI Write helped me know what parts of my writing I should improve*; (5) *MI Write helped me keep track of my progress in writing*; (6) *MI Write helped me become a better writer*. Cronbach's alpha for this subscale was 0.85, indicating high reliability. Accordingly, for each student, we calculated the average score of all six items and used this aggregate *usefulness scale score* in subsequent analyses.

### 2.4 Data analysis

#### 2.4.1 Latent Dirichlet Allocation (LDA)

We used LDA, a generative statistical model [28,29], in our study to extract latent topics comprising semantically related words from students’ comments. The primary advantage of the LDA approach is its ability to systemize the categorization of observations into topics, facilitating the establishment of possible themes in connection with the research context. It also provides researchers with an efficient means of analyzing large volumes of textual data and deriving meaningful insights at scale. In contrast to traditional qualitative methods, such as interviews or focus groups, which are time-consuming and subjective in data analysis, LDA automates the categorization and analysis of textual data, enabling a more systematic and streamlined approach to identifying themes and patterns (e.g., [[30], [31], [32]]). This quantitative framework allows for objective quantification of topic prevalence and relationships within the data, addressing the limitations associated with handling large datasets and ensuring replicability across different contexts or studies.

The identified topics do not tend to be self-evident (e.g., [40]), so interpretable LDA results often necessitate mixed methods efforts including quantitative scrutiny and qualitative interpretation. This combination of quantitative analysis via LDA with post-hoc qualitative interpretation enhances the richness and depth of the findings. As we have done in the present study, it is possible to explore the qualitative aspects of topics identified through LDA by examining specific excerpts or examples to gain a nuanced understanding of students’ perceptions. Also, it is possible to associate other sources of data—such as quantitative survey data—with the LDA results to further triangulate findings. This mixed-methods approach not only facilitates a comprehensive exploration of the data but also strengthens the reliability and validity of the findings.

To conduct the LDA analysis, we followed a standard procedure pipeline from data preprocessing, statistical modeling, identifying descriptors, to interpreting documents. We conducted data preprocessing and statistical modeling in Python via the Jupyter Notebook environment. Given the potential risk that topic models that perform better on held-out likelihood may infer less semantically meaningful topics (e.g., [40]), we analyzed LDA statistical results jointly with qualitative interpretation based on the context of our study. Indeed, the joint computational and interpretive necessity with respect to utilizing LDA has been increasingly recognized in educational research, such as coupling machine and theory-driven expert judgments of student perceptions of teaching behavior in secondary schools in the Netherlands [41], and validating computationally generated topics from student written feedback by interacting with qualitative interpretation and literature to improve the quality of teaching in Finnish universities [42].

#### 2.4.2 Data preprocessing

The first step of data preprocessing involved eliminating entries that were either empty or minimally informative (e.g., “I liked it” or “I don't know”) or irrelevant (e.g., “I want to go home” or “lol”) based on qualitative analysis. Specifically, the first and third authors independently reviewed all 981 comments, identifying 92 comments to be eliminated based on these criteria. Inter-coder agreement was 99 % and all disagreements were resolved by consensus.

The second step consisted of tokenization (i.e., dividing texts into token elements) and lemmatization (i.e., converting words to their base form), which were performed using SpaCy (https://spacy.io/), a robust open-source library for natural language processing in Python. The tokenization process involved the extraction of three most informative part-of-speech types (i.e., nouns, adjectives, verbs) to establish the basis of lexis for our corpus.

To preprocess the data further, we expanded the list of stop-words recursively using the default English language word list in SpaCy. For example, we removed general words related to our study such as write, MI, essay, and program; model verbs such as would, could, and may; reporting verbs such as find, think, and let; and general proper nouns such as people, thing, and stuff. The limited size of the analytic sample precluded the use of term frequency–inverse document frequency (TF-IDF) for constructing a bag of words model. TF-IDF penalizes words with high raw frequency based on the number of documents in which they appear [43], and is typically applied with much larger corpora (e.g., a Kaggle dataset of 50,000 wine reviews; [44]).

As an additional data preprocessing step, we dichotomized students’ MI Write usefulness scale score ratings into high and low categories. MI Write usefulness scale score ratings equal to or greater than 2.0 (i.e., falling in the range of “agree” to “strongly agree”) were categorized as *high usefulness,* while subscale ratings less than 2.0 were categorized as *low usefulness*. Dichotomizing in this way allowed for answering RQ2, which considered whether there were differences in topic emphases according to students’ usefulness ratings.

#### 2.4.3 Statistical modeling

We fit the *Gensim* LDA model to our corpus, which consisted of vectorized bag-of-words representations associated with their respective frequency counts for each student comment entry. As a rule, the evaluation of the appropriateness of an LDA model is informed by the ideal number of topics chosen, as indicated by coherence scores that reflect the semantic relatedness of highly influential words identified within the latent topics. Among multiple coherence measures (e.g., [45]), we utilized the *UMass evaluation score* [46], which uses the log probability of word co-occurrences, and the *CV score* [47], which uses the normalized pointwise mutual information and cosine similarity of the topic words. The coherence scores’ benchmark suggests that the preferred number of topics is indicated by a lower UMass score and a higher CV score, respectively. The size of the analytic sample meant that local rather than global minimum and maximum were appropriate (e.g., [44]).

#### 2.4.4 Interpreting topics: qualitative analysis

To interpret the identified topics, we first identified descriptors for these topics by examining the top ten most frequently occurring words within each topic and whether some of these key words occurred uniquely within a topic. Then, we performed a qualitative analysis to identify emerging themes. To do so, we identified the top fifteen user comments that registered the highest percentage contribution to each of the four latent topics, respectively. Then, using a thematic analysis (see [48]), the first and second authors collaboratively established consensus as to the interpretation of a given topic through an iterative process. Topic themes were interpreted in connection with, but not solely determined by, the identified key words for each latent topic.

## 3 Results

### 3.1 RQ1: main themes about MI write

**Fig. 1:**
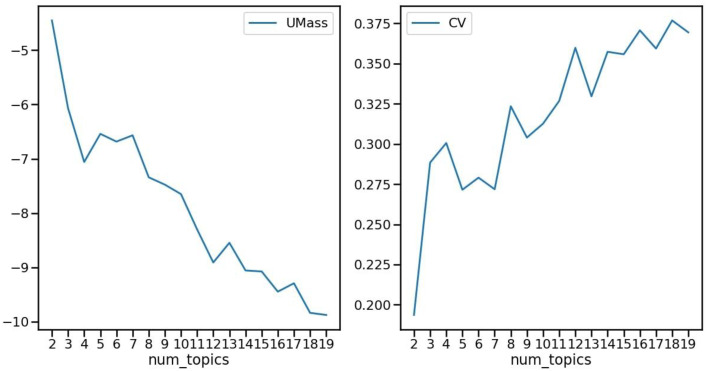
LDA coherence scores as evaluation metrics.

Results indicated that the optimal LDA model for our corpus consisted of four latent topics. As shown in Fig. 1, the UMass coherence score reached the local minimum −7.059 in the neighborhood of one to seven topics, and fairly consistently, the CV coherence score reached the local maximum 0.300 in the neighborhood of one to eight topics. Within these ranges, the convergence to a count of four topics by both metrics indicates the ideal number of topics to be fit into the optimal LDA model.

Based on this optimal, four-topic LDA model, we identified descriptors for these topics by examining the associated top-ten ranked words. Table 2 presents the key words and proportional representation with respect to the associated topic. For instance, the word “sentence” represented 1.5 % of all tokens within Topic 1; the word “website” accounted for 2.8 % of all tokens within Topic 2. The colored highlight indicates key words that are not only unique but informative for a given topic. For example, “word” and “sentence,” both of which are concerned with language-related issues, were distinctly identified for Topic 1, whereas “website” and “fix,” which are related to learning technology, were noticeable within Topic 2 only. These unique words speak to the idiosyncratic features of any themes arising from the corpus.

**Table 2:** Top ranked key words with proportional representation by topics.




Note. PR = proportional representation.

Furthermore, it is noticeable that certain key words such as “score,” “grade,” “creator,” “feedback,” and “improve” were identified across multiple topics. For instance, “score” has a probabilistic value of 0.038 in Topic 1, and 0.024 in Topic 4. Instead of removing such words so vocabulary associated with each topic was mutually exclusive, we retained them alongside the uniquely identified words for each latent topic. This decision aligns with the underlying assumption of LDA modeling, which posits that a corpus and its constituent words exhibit probabilistic relationships with respect to all topics. Indeed, the rankings of those identical words varied across topics, meaning they hold varying weights to what constitutes a particular topic.

Thus, the LDA modeling results motivated us to qualitatively explore four latent topics, which we did through consideration of their associated keywords and analysis of the top 15 comments most strongly associated with each topic—see Tables S1–S4 in the supplementary material that provide these comments. We present themes emerging from this qualitative analysis along with supporting quotations below.

#### 3.1.1 Topic 1: students desire more in-depth feedback

Across the sample, 31.23 % of students’ comments were most strongly associated with Topic 1. The distinct words identified by LDA modelling for Topic 1 included understand, system, word, and sentence. Considering these keywords along with the top 15 comments most strongly associated with Topic 1 (see Table S1 in the supplemental materials) led us to interpret Topic 1 as related to students’ desire for more in-depth feedback. Accordingly, many comments associated with Topic 1 were critical of MI Write.

Among the comments that most strongly aligned with Topic 1 were those expressing a desire for more in-depth feedback from MI Write. For example, one student contrasted its feedback to that of a teacher:

*The only thing that I find slightly valuable about MI Write is its scoring system, which gives me a general idea about the quality of my writing, but even with that I would prefer to ask a teacher and they could give me much more in-depth opinions and suggestions and direct me to what I did wrong.*

Students also expressed a desire for feedback that was both more clear and more localized. Students described MI Write's feedback as “too vague” or it “sometimes get me confused,” resulting in MI Write supporting “a very inefficient method of critiquing work.” Students desired to have a “direct answer” and suggested that MI Write “should tell a student in which places they can improve. For instance, an exact sentence which could be made better.” Another student suggested that MI Write be “more detailed,” such as by “adding an alternative sentence for the sentence that the student worded wrong; maybe introducing a word bank on the side to replace the words with more advanced words.”

Another student expressed frustration with both the automated feedback's lack of clarity and lack of direction it provided, and how these limitations did not enable the student to improve their knowledge or thought process:

*They say things like “organize better.” What does that mean? Yeah, thanks, I'll put my conclusion paragraph first. Its suggestions are vague and useless. So, instead of revising my work to get a better score, my classmates and I just tweak it randomly to try and get a better score. This isn't improving my thought process. It doesn't give a rubric for what argumentative essays should be like (even on state tests).*

Finally, comments in Topic 1 expressed frustration with the accuracy of MI Write's plagiarism and editing functionality. Students described the plagiarism detector as incorrectly flagging sources that they cited or quoted as well as “everyday words” and phrases. Similarly, MI Write's editing functionality was critiqued for “sometimes point(int) out misspelled words when they are not. I would write a name, and it points it out as wrong.”

Thus, Topic 1 identified students’ desire for more in-depth feedback that also was more localized, direct, and accurate.

#### 3.1.2 Topic 2: students desire an enhanced user experience

Across the sample, 25.64 % of students’ comments were most strongly associated with Topic 2. The distinct words identified by LDA modelling for Topic 2 included assignment, mistake, website, and fix. The predominant theme that emerged from the comments most strongly related to Topic 2 (see Table S2) was students’ desire for an improved user experience, with a particular focus on navigating the program and its visual presentation.

Students expressed a desire for usability enhancements related to navigating the program. As an example, a student proposed the idea of visually highlighting new assignments for better distinction:

*If a student is assigned a new assignment, it should pop up in a very noticeable way. A student might not remember to check their notifications for a new assignment, which can lead to them creating a different prompt to create their essay. This may contribute negatively to the person's grade.*

Another student suggested that MI Write could be improved by “making the options a little bit easier when I get on it. I don't directly know what to click or what to go on.”

Another salient aspect of students’ user experience appears to be MI Write's visual presentation. One student described how MI Write's aesthetics influenced their engagement:

*I personally think that the colors and logo of the website/program could be improved. I feel that the color scheme could be a bit brighter and welcoming. It seems a bit boring and too professional (especially since it is being used by youth). The dark blue and white somewhat turns me away.*

Similarly, another student desired MI Write to be “more fun and appealing.” This student felt that MI Write was “boring to go on” and preferred to use Google Docs because they could “edit the page and make it more aesthetically pleasing; it makes me motivated. MI write does the opposite.” These students’ comments highlighted that AWE's aesthetics and design can have an associated effect on the motivation of its users.

Students’ comments about the visual presentation and usability of MI Write also extended to how MI Write's feedback was presented. Whereas comments in Topic 1 highlighted students’ desire for more in-depth, direct, and accurate feedback, comments in Topic 2 pertaining to feedback focused on desiring visual or other system triggers/reminders to revise. For example, one student commented that “most of the time, when I click ‘revise essay’, I forget what I need to fix in it. Just a little notification or a little reminder that tells me what I need to revise would be really helpful.” Another student felt that simultaneous feedback—rather than feedback after submitting a draft—would enhance the efficiency and effectiveness of their writing. The student recommended to “give students hints or ideas for better ways to fix their writing, like paraphrasing. Not to be done after the students submit the assignment, but while they're writing, so they could see their mistakes beforehand.”

Thus, Topic 2 identified students’ desire for an enhanced user experience, both in terms of ease of use and aesthetics.

#### 3.1.3 Topic 3: students value MI write as a learning tool but desire greater personalization

Across the sample, 27.44 % of students’ comments were most strongly associated with Topic 3. The distinct words identified by LDA modelling for Topic 3 included good, change, and hard. The word “good” registered the highest occurrence of tokens for Topic 3, accounting for 9.5 % of the total tokens. The theme most prominent in the comments related to Topic 3 (see Table S3) was that students valued MI Write as a learning tool but expressed a desire for greater personalization to enhance their user experiences.

Positive comments provided by students regarding AWE acknowledged the overall quality and effectiveness of the tool in supporting their writing. For instance, students said, “MI Write is a very good program that helps students with their writing, including myself” and “I think MI Write creators may want to know that people like me do not write well but MI Write has helped me write better essays.” Another student wrote, “I want the MI Write creators to know that I love the PEG.” Additionally, one student specifically praised the tool's usefulness in facilitating the writing process, stating “I believe that MI Write is a good program to help students of my age improve their writing and also a program that takes us, the students, through the writing process which guides us to becoming better writers.”

Nevertheless, while students generally found MI Write to be a beneficial learning tool, comments associated with Topic 3 identified some unique areas of personalization not represented in the prior topic areas. One of these areas was the age-appropriateness of MI Write's interactive skill-building lessons. One student described them as seeming “to be more for kids around the 2nd and 3rd grade, not 7th grade,” and felt that “it just babies down what does not have to babied down.” Another student commented on the age-appropriateness of the graphics in the lessons, writing “the weather and animal things made zero sense to me.” Students seemed to feel the program was not targeted to their age level.

A second area related to students’ desire for MI Write to better accommodate their diverse languages and registers. For instance, one student stated, “I wish it was more open to diverse language,” which was echoed in the comment of another student who desired MI Write to “integrate a more up-to-date vocabulary and a wide selection of slang” because that “would be more inclusive for dialogue and some first-person narratives.” Still another student wrote, “I feel with MI Write the writer is forced to write their essays in a very serious tone which could be hard and more challenging for some writers.” Finally, one student explained how MI Write's scoring would penalize use of non-traditional language and vocabulary:

*Sometimes the grading would take points for something you wanted to be in the passage. For example, I would have one of my characters speak in an accent and would get marked off for that. Other than this, MI Write is wonderful.*

Thus, Topic 3 captured students’ overall positive perceptions of MI Write along with an expressed desire for increased personalization, especially related to its age-appropriateness and inclusivity of diverse languages and registers.

#### 3.1.4 Topic 4: students desire increased fairness in automated scoring

Among the sample, 15.68 % of students’ comments were most strongly associated with Topic 4. The LDA modeling identified distinct words for Topic 4, such as support and app. Contrary to the expected focus of these distinct words, the dominant theme that emerged from the comments centered around students’ desire for increased fairness in automated scoring, with frequent mentions of “score,” “grade,” and “scoring” among the top 15 comments associated with this topic (see Table S4).

Students commented on two main areas where fairness in automated scoring could be improved: recognition of diverse language and appropriately cited text. Considering sensitivity to diverse language, which was also reflected in Topic 3, one student wrote that, “it does disappoint me when it grades because it does not grade fairly. When I write a story or piece of text, it grades my characters’ names as a low score which affects my total writing score.”

Regarding citations, students frequently mentioned MI Write's failure to identify appropriate uses of source material as a significant factor affecting the fairness of the automated scores. A student mentioned, “sometimes during the scoring process, text evidence is counted as plagiarism even though it has been properly cited.” Student comments expressed a strong desire to enhance the design of MI Write to enable intelligent recognition of textual borrowing, which was also identified in some comments associated with Topic 1, though the linkage to scoring was much stronger here in Topic 4. For example, one student commented:

*The scoring is sometimes annoying because it can say you plagiarized, even when you did not. Then when you check the source it claims you plagiarized, it is not even related to the phrase. Then it scores you really low which is sometimes frustrating.*

These two areas—recognition of diverse language and appropriately cited text—came together in one student's comment critiquing the fairness of MI Write's scoring, which read, “when I quote stuff and it uses slang like in a poem, it decreases my average points because it's not a proper grammar.” The student then suggested that MI Write “add like a feature where if you were to quote things, it won't affect the student's average score.”

Finally, one comment perfectly articulated the underlying theme of Topic 4 as pertaining to students expressing a desire for increased fairness in automated scoring. The student wrote, “I also believe that writing contains depth between the writer and the reader, and a simple score does not represent writing as a whole.” Indeed, other students commented that, “I would like the creators to develop a more accurate scorer because sometimes I feel like I deserve a better score out of what I wrote,” and “sometimes, even when an essay is [written] perfectly well, MI Write will give the essay a lower score than it actually should be.”

Thus, Topic 4 identified students’ desire for increased fairness in automated scoring, especially as it pertained to recognition of diverse language and use of source materials.

### 3.2 RQ2: theme differences based on students’ perceptions of usefulness

**Fig. 2:**
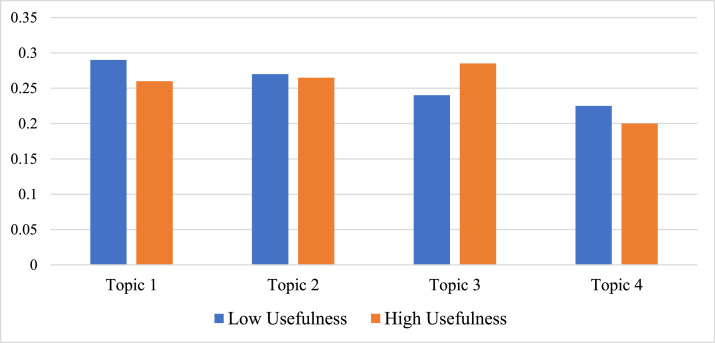
The distribution of topics by low and high usefulness ratings.

Fig. 2 displays the distribution of the four topics separately for student groups with overall low usefulness ratings and high usefulness ratings.

Fig. 2 shows that students who perceived MI Write to have overall low usefulness expressed a higher proportion of comments associated with Topic 1 (29 %) than students who perceived MI Write to have overall high usefulness (25 %). The reverse was true for Topic 3: this topic was more strongly evident among comments of students who reported high usefulness (28 %) than among those who reported low usefulness (24 %).

These findings help to further corroborate our qualitative interpretation of the topics presented earlier. As we described, Topic 1 appeared primarily concerned with students’ keen demand for in-depth feedback. This would be characteristic of learners likely to report that MI Write lacked sufficient usefulness to meet those demands. Further, Topic 3 was characterized by generally positive comments about MI Write, which would be expected from students who had more positive perceptions of its usefulness.

There were no distinct differences between student groups for Topic 2: these accounted for 26 % of the proportion of comments for both the low usefulness and high usefulness student groups. Finally, students who rated MI Write's usefulness low were only slightly more likely to offer comments associated with Topic 4 (22 %) than students who rated MI Write's usefulness high (20 %). This finding further corroborates our earlier qualitative interpretation of Topic 4, as its theme focused on students’ desire for more accurate scoring, especially when students cited sources.

## 4 Discussion

The present study applied LDA topic modeling to examine middle school students’ perceptions of the AWE system MI Write, including their suggestions regarding its overall functionality and specific features. Through the LDA modeling, we identified four latent topics, which included: (1) students desire more in-depth feedback; (2) students desire an enhanced user experience; (3) students value MI Write as a learning tool but desire greater personalization; and (4) students desire increased fairness in automated scoring. Results showed that while topics were relatively distinct, they were not entirely mutually exclusive. In some cases, topics reinforced each other, but with some variations in perspectives or domains. A detailed reading of the user comment data enriched our understanding of the topics based on the identified key words. A follow-up analysis indicated that the distribution of the four topics covaried with students’ overall ratings of MI Write's usefulness, with some topics being more visible for one group than the other.

### 4.1 RQ1: main themes about MI write

Our results showed that students desire more in-depth, direct, actionable, and accurate feedback (Topic 1), which closely aligns with the findings of previous research that has largely focused on undergraduates in EFL or ESL contexts [16,17,21]. Consistent with these findings, the U.S. middle school students in the present study expressed dissatisfaction with perceived superficiality of the automated feedback, as they believed it did not contribute to the enhancement of their thinking abilities and processes, which are predominantly associated with higher-level writing skills.

We further learned that middle school students desire an enhanced user experience (Topic 2), which builds upon and extends findings from previous research. For example, Li et al. [21] reported that ESL undergraduates with lower language proficiency levels appreciated AWE systems that visually highlight areas requiring improvement in content and organization. This feature alleviates the difficulty of understanding potentially complex instructional language used in AWE systems. In the present study, students provided quite broad recommendations for improved visual representations, including incorporating diverse color schemes and aesthetically pleasing website interface designs. Importantly, students in our study described the influence of aesthetics on their engagement and motivation. This finding addresses a gap in previous research [[12], [13], [14], [15]], which primarily focused on studying the associated effects of AWE on writing motivation without considering specific stimuli or influential factors associated with the AWE system per se. Furthermore, students’ desire for specific, higher-level feedback aligned with previous studies. However, a notable addition was the prominent request for simultaneous feedback and revision in writing, aiming to improve learning efficiency and effectiveness. In the U.S., middle school students regularly use GoogleDocs in school, a program that allows for receiving real-time feedback from peers or their instructor. Perhaps their familiarity with GoogleDocs—indeed, students mentioned GoogleDocs in their comments (see Tables S1–S4)—or other contemporary educational technology tools led students in the present study, rather than in past studies, to highlight this desire for simultaneous feedback/revision capabilities within AWE.

Findings additionally showed that U.S. middle school students valued MI Write as a learning tool (Topic 3), which aligns with findings of prior research showing that students generally hold positive perceptions towards AWE [14,15] and specifically MI Write [[12], [13]]. However, a further component of this finding—and one not previously emphasized in research—was students’ specific requests that MI Write better accommodate diverse languages, accented languages, and slang. This request may not have been surfaced in prior research on ESL/EFL undergraduates because those students are trying to master standard, academic English, whereas U.S. middle school students write for other purposes in English, including expressing their creativity. Moreover, the present sample was highly diverse in terms of race and ethnicity, which more likely raised concerns about dialect recognition among the sample.

Results also exposed students’ perceptions of fairness of AWE scores, particularly concerning the recognition of individual authorship through distinct language features and textual borrowing techniques (Topic 4). While previous studies have examined general perceptions of fairness in AWE scoring [[14], [17], [49]], our findings expand this literature by revealing specific factors that can compromise students’ perceptions of the validity of automated scoring. Concerns raised about AWE scoring, particularly regarding textual borrowing that encompasses authors’ language styles, tasks, and strategies are unique contributions of the present study. Perhaps this also reflects updated instructional priorities in the U.S. relative to the past when source-based writing was not as highly emphasized or frequent. In the context of AWE evaluation, the key challenge lies in fine-tuning the AWE scoring system to be responsive to the diverse textual borrowing needs and practices of users.

### 4.2 RQ2: theme differences based on students’ perceptions of usefulness

The student group that perceived the AWE system to be relatively less useful showed a stronger inclination towards demanding in-depth feedback and linguistic support (Topic 1). On the other hand, the student group that perceived AWE to be relatively more useful exhibited a greater likelihood of commenting on MI Write's utility while concomitantly desiring greater opportunities for personalization, particularly with respect to a liberal use of languages (Topic 3). This preliminary finding provides a basis for further investigation through confirmatory data analysis in future studies. By delving deeper into these relationships, a more comprehensive understanding of the nuanced dynamics between AWE system perception, feedback preferences, and individual authorship may be achieved.

### 4.3 Limitations

Results of the present study should be interpreted considering several limitations. The first set of limitation relates to the LDA methodology employed. Although the diagnosis of LDA coherence scores is stable in that it recursively produced four topics as the optimal number of topics, variations will occur for the keyword output with each run of LDA modeling. Also, the rankings of key words with respect to their proportional representation within a topic may fluctuate. To mitigate this latter possibility, we reran our LDA modeling a second time and confirmed that a large proportion of identified keywords overlapped with preliminary results. Moreover, keywords generally retained their proportional representation within a topic. Nevertheless, it is important to note that slight differences in keyword prevalence by topic may point to relatively different perspectives or concerns. This limitation underscores the importance of triangulating the LDA output with results of qualitative analysis.

A second limitation relates to our decision not to apply TF-IDF given the limited scope of the analytic sample. Specifically, our corpus included 893 student responses with an average length of 20.43 words. The corpus size introduced a risk that the TF-IDF algorithm, which assigns more weights to words with lower frequency in a document, might identify idiosyncratic features trivial enough to disguise the impact of larger topics from our corpus. Nevertheless, to address potential concerns regarding this decision, we reran the LDA model while applying TF-IDF. Results generated essentially the same key words as those reported in the results, though the ranking of the keywords changed slightly. Nonetheless, the proportional presentation with respect to a topic for those keywords normally fell between 1 % and 4 %, so any difference in ranking did not seem to be influential in terms of how we interpreted the results. Overall, results appeared robust whether or not TF-IDF was used.

In addition, it is important to note that we sampled from a population of students with no prior experience using MI Write, thus constituting initial users. Initial users might have different expectations compared to those with more experience, gained from prolonged usage over multiple academic years. Nevertheless, we believe it is a strength of our study to have sampled from a population of middle school students with no prior experience using MI Write. Had we sampled from experienced users, that may have introduced a self-selection bias, since those with positive initial experiences would be expected to continue using MI Write, while those with negative initial experiences would not be expected to continue. Sampling initial users as we did arguably allows for understanding a greater diversity of student perceptions.

## 5 Conclusion

Our research significantly advances the field by addressing critical gaps in the understanding of students' perceptions of AWE, specifically MI Write. While prior studies have delved into the impact of AWE on writing outcomes, few have conducted an in-depth exploration into the nuanced perceptions and experiences of middle school students in the U.S. context. Our study stands out by not only focusing on this underrepresented demographic but also by employing a pioneering analytic method—latent Dirichlet allocation (LDA). This approach has enabled us to uncover latent themes from students' comments, offering a richer and more comprehensive perspective than traditional methods. Our findings, derived from a diverse sample of U.S. middle school students, provide insights that pave the way for the creation of AWE systems that are more attuned to the needs and preferences of diverse student populations, ensuring a more inclusive and effective learning experience. Furthermore, by demonstrating the potential of LDA in this research context, our study sets a precedent for future investigations, promoting methodological innovation in the domain.

Relevant to researchers and educators, students’ desire for more in-depth feedback underscores the necessity of implementing AWE alongside teacher feedback (e.g., [[12], [50]]) or feedback from peers [51,52]. Although AWE's capability of providing personalized and high-quality feedback surely will improve in the coming years given rapid advancements and applications of large language models in education [53], human feedback is critical for providing the nuanced guidance, personalized support, and holistic evaluation that students need. As students’ comments indicate, AWE can serve as a valuable tool to augment and enhance the feedback process, but AWE can never replace human feedback (nor should it). Thus, it behooves researchers and educators to identify and share effective examples of integrating AWE within teacher-led instruction.

For AWE developers, the identified topics in this study highlight areas for future development to enhance AWE functionality. Students’ desires for an improved and more personalized user experience together with increased fairness in automated scoring could be addressed through advancements in various aspects of AWE design. This includes improving the visual aesthetics and navigational aspects of the system to create a more motivating and user-friendly learning environment.

Further, incorporating diverse language and registers, as well as increasing the accuracy of the automated scoring system and its recognition of source use, would contribute to a more inclusive and fair AWE experience for students. Yet, these requests challenge conventional beliefs about what constitutes good writing, introducing complexity and dynamics to the legitimacy of AWE feedback and scoring. Indeed, expanding AWE to be more inclusive of dialect and slang will necessarily involve some trade-offs among scoring accuracy, feedback specificity, and inclusiveness, as expanding AWE training data to include more varied use of language including slang expands what “good writing” is. One option may be for AWE systems to afford teachers greater control over the automated scoring and feedback, allowing them to select whether the system will flag uses of slang and dialect.

There remains a strong need to identify effective and scalable writing interventions, especially among diverse U.S. middle school students. As the field of AWE continues to evolve, driven by advancements in machine learning and artificial intelligence technologies, the insights gained from this study should not only inform the implementation and improvement of existing AWE systems but also guide the development of future generations of AWE technology to hopefully meet this need. To this end, ongoing research should delve deeper into students’ perspectives of AWE, contributing to a continuous cycle of validation and development aimed at enhancing the usability, usefulness, desirability, and overall effectiveness of AWE in supporting writing instruction and learning.

Finally, the present study exemplifies the utility of LDA in facilitating such future research. Conducting in-depth qualitative analysis, especially with large sample sizes, can be inherently time-consuming, difficult to replicate, and subjective. LDA, offers a systematic approach to uncover latent topics and patterns within the data, allowing for a more comprehensive and replicable understanding of students' perceptions and desires regarding AWE. Its application in this study as part of a robust mixed-methods analysis underscores LDA's potential as a valuable tool for researchers seeking to explore students’ view of AWE. Thus, we hope our study motivates other researchers to consider applying LDA to further explore students’ perceptions of AWE.

## CRediT authorship contribution statement

**Joshua Wilson:** Conceptualization, Methodology, Formal analysis, Investigation, Writing – original draft, Writing – review & editing, Supervision, Project administration, Funding acquisition. **Saimou Zhang:** Conceptualization, Methodology, Formal analysis, Investigation, Writing – original draft. **Corey Palermo:** Resources, Writing – review & editing, Supervision, Project administration, Funding acquisition. **Tania Cruz Cordero:** Investigation, Writing – review & editing. **Fan Zhang:** Formal analysis. **Matthew C. Myers:** Investigation. **Andrew Potter:** Investigation. **Halley Eacker:** Investigation, Resources, Supervision, Project administration. **Jessica Coles:** Investigation, Resources.

## Declaration of competing interest

The following authors declare no conflicts of interest relevant to this work: Joshua Wilson, Saimou Zhang, Fan Zhang, Tania Cruz Cordero, Matthew C. Myers, Andrew Potter. The following authors are or were employed by Measurement Incorporated at the time the research was conducted: Corey Palermo, Halley Eacker, Jessica Coles.

During the preparation of this work the authors used ChatGPT in order to revise portions of the text for clarity and succinctness. After using this tool, the authors reviewed and edited the content as needed and take full responsibility for the content of the publication.

## Appendix Supplementary materials

Image, application 1
