# Supplementary material for: A Latent Dirichlet Allocation approach to understanding students’ perceptions of Automated Writing Evaluation
Source: Comput Educ Open. 2024 Jun;6:None. doi: 10.1016/j.caeo.2024.100194 (PMC11212450; doi:10.1016/j.caeo.2024.100194)
Supplement: Supplementary file 1 [file mmc1.docx]

**Table S1 (40% Usefulness = 1; 59% overall)**

*Top Fifteen Comments with the Highest Percent Contribution for Topic 1*

| ID | Percent contribution | Usefulness Rating | Comment |
| --- | --- | --- | --- |
| 659 | 0.9739 | Low | MI write could use a LOT of help. Basically, it looks horrible and is difficult to use. It looks like it was a website made in the 90s. It's hard to use and poorly designed. Your plagiarism formula is trash. It links sources and then when you cite them it flags you for plagiarism. Knowing how much of your essay is evidence is helpful but it should not be marked as plagiarism.  I recommend a grammar check or synonym suggestion that you can use during your writing process. I also think your suggestion or criticisms are useless. They say things like "organize better". What does that mean? Yeah, thanks I'll put my conclusion paragraph first. Your suggestions are vague and useless. So instead of revising my **work** to get a better **score**, my classmates and I just tweak it randomly to try and get a better **score**. This isn't improving my thought process. It doesn't give a rubric for what argumentative essays should be like (even on state tests).    I am a creative person. There is nothing unique that I can include in my writing. I find myself struggling to connect with my reader when using your formula. What is the point of writing if you can’t connect with the person reading your **work**? Personally, I think writing has to be graded by humans but if my **work** is going to be graded by a robot, might as well have the robot be good at its job. |
| 49 | 0.9639 | Low | Personally, I feel that MI Write is another complicated teaching tool. It is not only complicated for the students, but it also **adds** another platform that teachers must monitor and use. From the student’s perspective it doesn't really provide any unique tools that cannot be figured out on a pen and paper, or other tools already provided. Its writing function isn't really unique it just seems like another document writing application. The only thing that I find slightly valuable about MI Write is it's scoring **system** which gives me a general idea about the quality of my writing, even with that I would prefer to ask a teacher and they could give me much more in-depth opinions and suggestions and direct me to what I did wrong. While I am not a teacher, I do **understand** the general stresses and difficulties of teaching, and I feel its unnecessary to **add** another platform for teachers to use when Google Docs is an alternative on the same or some would say higher level. |

| ID | Percent contribution | Usefulness Rating | Comment |
| --- | --- | --- | --- |
| 651 | 0.9601 | Low | I believe that the user interface is horrible. The website looks like it was designed in 1987 and was never updated again. It is very difficult to find what you need to find.  Along with this, the criticisms are all-around horrible. Whenever I quote an article and cite my source, it marks my **work** as "plagiarism" and lowers my **score**. Also, the criticisms that the program gives me are very vague. It always gives me the same 4 questions like "Are your paragraphs and **sentences** organized correctly?" This does not help at all and is too vague for me to even **understand** what they are trying to say. If I want to **improve** my essay, I have to read a 3-page article about organizing **sentences** (which is also very vague), and then try to find which **sentence** was wrong in my essay. It is just a very inefficient method of critiquing **work**, and I don't like it at all. |
| 598 | 0.9194 | Low | MI Write's website design is unappealing and difficult to use. It plagiarized many phrases that were everyday **words**. I disliked how difficult it was to fix your drafts. I was confusing knowing which draft you were on or how to revise it. The comments where pretty helpful but a lot of the times were very vague. |
| 639 | 0.8983 | Low | I feel like maybe a more detailed grading **system** would help students fix their writing and **improve** along the way, maybe **add** an alternative **sentence** for the **sentence** that the student **worded** wrong; maybe introduce a **word** bank on the side to replace the **words** with more advanced **words** this would help the student have a better perception and a more open mindset to a new set of **words**. |
| 652 | 0.8964 | High | MI Write is a fantastic program that really helps me plan out my writing and **score**. However, I do have a suggestion. When I have turned my writing in, it sometimes points out misspelled **words** when they are not. I would write a name, and it points it out as wrong. Other than that, I have no concerns. Thank you for providing students with a fantastic program like MI Write. |
| 702 | 0.8933 | High | When using MI Write there are many elements of it that fascinated me while others needed some help. I love the fact it gives you a **score** at the end while telling you what you did wrong so students like me can better **understand** our mistakes. On the other hand, I dislike how MI Write sometimes states you misspelled a **word,** but the **word** isn’t misspelled. If anything, I would say it’s the only flaw in the whole program, it’s very well liked by my classmates and me. |
| ID | Percent contribution | Usefulness Rating | Comment |
| 640 | 0.8932 | Low | MI Write is a good program however, I don't believe it gives **scores** that are fair to everyone. For example, someone might write an essay with many **words**, and another that has a lot fewer **words**. The person with more **words** gets more points, while the person with few **words** gets less points even though they did every direction asked. |
| 723 | 0.8866 | High | I'd like the MI Write creators to know that they can implement **systems** to help with user-friendliness, such as a separate tab on MI Write for peer reviews and an easier to **understand** scoring chart, listing what you can fix at a list on the top of the grading chart. |
| 667 | 0.8805 | High | Here is my idea for MI Write. Instead of using a robot to check your writing and **score** it, how about the teacher of the class can check your writing and **scores** it, because I believe the robot doesn't **score** correctly unlike a teacher who reads it. |
| 564 | 0.8713 | Low | Make your "Anti-Cheating' **system** better. Many students lose hours of **work** due to the anti-cheating **system** going off for a silly reason. |
| 320 | 0.8592 | Low | to not having to rewrite the whole paragraph because I misused a **word** and certain section because it does not really teach you much it just tells you that even though you used the correct **word**, it not "used properly" which can be sometimes pretty annoying. |
| 374 | 0.8585 | High | I think that revising the grading **system** would be very beneficial, as on one of my writing projects went from low eighties to the nineties after **adding** 2 simple **sentences** to my ending paragraph. |
| 40 | 0.8548 | Low | The grading **system** is fairly nice, but I believe they should tell a student in which places they can **improve**. For instance, an exact **sentence** which could be made better. |
| 851 | 0.8542 | High | The Feedback sometimes gets me confused. It asks me questions about how I did my writing, but I find it easier when I get a direct answer like If I need check out how I transition my **sentences** |

*Note*. Keywords for Topic 1 are in bold, including word, score, improve, understand, work, system, sentence, easy, add, organizer.

**Table S2 (60%; 66% overall)**

*Top Fifteen Comments with the Highest Percent Contribution for Topic 2*

| ID | Percent contribution | Usefulness Rating | Comment |
| --- | --- | --- | --- |
| 224 | 0.9267 | High | I personally think that the colors and logo of the **website**/program could be improved. I feel that the color scheme could be a **bit** brighter and welcoming. It seems a **bit** boring and too professional (aseptically since it is being used by youth). The dark blue and white somewhat turns me away. Maybe a lighter blue and another color would work better. But that's my personal opinion. |
| 872 | 0.9175 | High | If a student is assigned a new **assignment**, it should pop up in a very noticeable **way**. A student might not remember to check their notifications for a new **assignment**, which can lead to them creating a different prompt to create their essay. This may contribute negatively to the person’s grade. |
| 761 | 0.9000 | High | Maybe you guys could add something where when you go to revise your essay and **fix** **mistakes** it gives you a little **bit** of advice on how to make that certain type of essay better (from what they wrote already). Rather than just some spelling **mistakes** you can **fix.** I like these too, but some minor advice would help too. |
| 801 | 0.8964 | High | Nothing really, but other than reminding me what I need to **fix** on an essay would be **great**. Most of the time, when I click revise essay, I forget what I need to **fix** in it. Just a little notification or a little reminder that's tells me what I need to revise would be really helpful. |
| 491 | 0.8689 | High | The program MI writes is really supportive of kids writing and it's really **easy** to understand MI writes, it can be better by making the options a little **bit** easier when I get on it. I don't directly know what to click or what to go on. But after all, it's an amazing site when it comes to helping in reading and writing. |
| 631 | 0.8635 | High | TIME LIMITS!! Some students may not realize there is a time limit. Yes, you can go back still and revise it. Although, some students in my class get a huge fright and have to tell the teacher about the sudden occurrence. After all, the program is absolutely **great**. |
| 6 | 0.8583 | Low | I think that the grading and revising should be a little **bit** more realistic because it doesn’t always notice spelling **mistakes** and punctuation. |
| 612 | 0.8550 | Low | Make the **website** more fun and appealing its boring to go on I usually just go to GoogleDocs because I can edit the page and make it more aesthetically pleasing it makes me motivated. MI write does the opposite. |
| ID | Percent contribution | Usefulness Rating | Comment |
| 304 | 0.8481 | Low | I think it would better if they were to make a kind of guide to assist starting your essay. As for me personally I struggle at starting as I can’t come up with things on the spot as I am not very creative. |
| 625 | 0.8366 | High | Students like me have creative minds and I feel as though MI Write might restrict them from it. Whenever I try to write in my style, it tries to correct some of the writing which was the parts that I added my style in it. |
| 170 | 0.8160 | Low | It’s way too complicated for me and others to use. Being told we were given a MI Write **assignment** was dreadful and soul sucking. |
| 375 | 0.8135 | Low | I feel as if the editing can be difficult as when you are editing, it doesn't let you look at your **mistakes**. I feel as if more tasks about modern things can help us be more engaged. |
| 695 | 0.8128 | High | What I would like the MI Write creators to know about how the program can better support students like me is to give students hints or ideas for better **ways** to **fix** their writing, like paraphrasing. Not to be done after the students submit the **assignment**, but while their writing, so they could see their **mistakes** beforehand. |
| 533 | 0.8028 | High | To make the prompts a **bit** more flexible and not stuck to one particular subject. |
| 498 | 0.8023 | Low | I would like the MI write creators to know that the feature where it corrects your grammar helps me understand my **mistakes** and not make them again. MI write helps me revise my work. |

*Note*. Keywords for Topic 2 are in bold, including easy, mistake, website, great, assignment, way, feedback, bit, fix, example.

**Table S3**

*Top Fifteen Comments with the Highest Percent Contribution for Topic 3*

| ID | Percent contribution | Usefulness Rating | Comment |
| --- | --- | --- | --- |
| 206 | 0.9249 | Low | It sucks make it better pls, I need several military trainings just to find out what assignment I have to do, as well as the lessons seemed to be more for kids around the 2nd and 3rd **grade**, not 7th **grade**. I feel like it just babies down what does not have to be babied down. There's a lot more issues but personally I don't **want** to write a college level essay for feedback on a site for school purposes. |
| 69 | 0.8933 | High | On certain occasions, it can be **hard** to find the right assessment. Also, when given a bad/alright score it would be nice to see where I went **wrong** because right now it just shows how. If I get a bad rating in organization, I would like to see where my organization was lackluster. |
| 692 | 0.8710 | Low | I **want** the MI Write **creators** to reconsider the timer that can be put on some assignments. Though many educators likely use this for timed writing exercises, students like myself may feel stressed as we write due to the pressure to finish before the timer runs out. |
| 576 | 0.8617 | High | I don't know if it's possible but maybe integrating a more up to date vocabulary and a wide selection of slang would be more inclusive for dialogue and some first-person narratives. |
| 844 | 0.8529 | Low | I think its pretty **good** from what I've seen and worked with over the past school year. It's efficient but sometimes needs explaining from an adult. Overall, its **good** though. |
| 553 | 0.8508 | High | MI Write is a very **good** program that helps many students with their writing, including myself. The only thing I would **change** is that sometimes the grading would take points for something you **wanted** to be in the passage. For example, I would have one of my characters speak in an accent and would get marked off for that. Other than this, MI Write is wonderful. |
| 615 | 0.8457 | High | I think MI Write **creators** may **want** to know that people like me do not write well but MI Write has helped me write better essays. I still do not believe the scores are on point because it is **hard** to believe I get **good** scores for essays so just find a way to boost students’ confidence. |
| 589 | 0.8236 | High | I **want** the MI Write **creators** to know that I love the PEG, but I wish it was more open to diverse language. |
|  |  |  |  |
| ID | Percent contribution | Usefulness Rating | Comment |
| 255 | 0.8062 | High | I **want** the MI write **writers** to know that I think it is **hard** to navigate throughout the website. And the technology for grading should be **changed**. |
| 559 | 0.8057 | Low | I feel with MI Write the **writer** is forced to write their essays in a very serious tone which could be **hard** and more challenging for some **writers**. |
| 275 | 0.8017 | High | It can make fails red instead of green because I think green means right. |
| 822 | 0.8002 | High | The MI write **creators** should know that we like the fun themes about stuff. We like to write about fun and exciting stuff that makes us **want** to write. |
| 480 | 0.7879 | High | In my opinion, I believe that MI Write is a **good** program to help students my age improve their writing and also a program that takes us, the students, through the writing process which guides us to becoming better **writers**. |
| 218 | 0.7850 | Low | The lessons were a nuisance, they felt useless. The weather and animal things made zero sense to me. |
| 348 | 0.7800 | Low | That some words that are actually spelled correctly get marked **wrong** and its pretty frustrating when it comes to getting a bad score because of them. MI Write its an okay space for writing but honestly, I’m not big fan of it |

*Note*. Keywords for Topic 3 are in bold, including good, change, improve, want, grade, easy, wrong, hard, creator, writer.

**Table S4**

*Top Fifteen Comments with the Highest Percent Contribution for Topic 4*

| ID | Percent contribution | Usefulness Rating | Comment |
| --- | --- | --- | --- |
| 646 | 0.9139 | Low | MI Write is an excellent app for writing, but I take it as it is a doc. I do not see the need to use it since it's like every writing program I write in, and it does disappoint me when it grades because it does not grade fairly. When I write a story or piece of text, it grades my characters' names as a low score which affects my total writing score, I also do not understand the chart that is given at the end, it does not explain how my writing is exactly. |
| 489 | 0.9121 | Low | One issue in the MI Write program is the computer-based grading recognized text-based evidence as plagiarism. I also believe that writing contains depth between the writer and the reader, and a simple score does not represent writing as a whole. |
| 666 | 0.9023 | High | When using the MI Write program, quoting stuff may be difficult for some students. When I quote stuff and it uses slang like in a poem, it decreases my average points because it’s not a proper grammar, so maybe you could add like a feature where if you were to quote things, it won’t affect the student’s average score. |
| 95 | 0.8731 | Low | The scoring is sometimes annoying because it can say you plagiarized, even when you did not. Then when you check the source it claims you plagiarized, it is not even related to the phrase. Then it scores you really low which is sometimes frustrating. |
| 526 | 0.8415 | High | I'm not sure if this is fixed yet but I would love it if copying and pasting textual evidence were allowed instead of having us type it out. |
| 112 | 0.8382 | Low | I think the MI write **creators** should get rid of the timer feature because, with the timer on the side, students may rush to complete the writing instead of taking their time. |
| 777 | 0.8374 | High | My teacher puts our percentile rank as our final **grade** in the gradebook, so I think it should give us an actual **score** instead of a percentile. |
| 318 | 0.8289 | High | Dependent on if the student is a first timer or not, think about the formality in language that you use, (not specified towards me) I have noticed that some students don't find it very comfortable to be very formal |
| 492 | 0.8256 | High | Sometimes in the **scoring** process it counts text evidence as plagiarism while it isn't since you cited the evidence from the text. |
|  |  |  |  |
| ID | Percent contribution | Usefulness Rating | Comment |
| 769 | 0.8219 | High | I would like the MI Write **creators** to maybe to possibly add more accuracy and maybe some bluffs to the **scoring** when it comes to writing essays. Sometimes, even when an essay is perfectly well, MI Write will give the essay a **lower** **score** than it actually should be. |
| 665 | 0.8202 | High | It would be better if you added more tips on how to be a better writer so that in the future the students could be a better writer for their future English teachers |
| 212 | 0.8136 | Low | I wish MI Write would actually give me pointers and tips to better my writing. Telling me more about my writing and giving me **feedback** would help me and many others in my situation. |
| 885 | 0.8116 | High | I would like the **creators** to develop a more accurate **scorer** because sometimes I feel like I deserve a better **score** out of what I wrote however, I know it can be very difficult to program things like that. |
| 450 | 0.8083 | High | I feel like the MI write program can make it better for student but have a certain button that say extra help just in case the students are stuck on something on the program or just need more explaining and Assistance. |
| 672 | 0.8018 | Low | To **improve** the **feedback** given after analysing writing, as it remains rather banal and unhelpful. As for the **scoring**, it seems MI Write is more **supportive** of essays and texts worded informally. |

*Note*. Keywords for Topic 4 are in bold, including support, score, feedback, grade, creator, improve, scoring, app, learn, low.
